# The nature, characteristics and associations of care home staff stress and wellbeing: a national survey

**DOI:** 10.1186/s12912-017-0216-4

**Published:** 2017-05-08

**Authors:** Muhammad Saiful Islam, Christine Baker, Peter Huxley, Ian T. Russell, Michael S Dennis

**Affiliations:** 1 Swansea University Medical School, Swansea University, Wales, UK; 2 Centre for Mental Health and Society, Bangor University, Bangor, Gwynedd, Wales UK

**Keywords:** Care home, Dementia, Care staff, Stress, Wellbeing, Job content, Job satisfaction, Productivity

## Abstract

**Background:**

The majority of residents in care homes in the United Kingdom are living with dementia or significant memory problems. Caring in this setting can be difficult and stressful for care staff who work long hours, have little opportunity for training, are poorly paid and yet subject to high expectation. This may affect their mental and physical wellbeing, cause high rates of staff turnover and absenteeism, and affect the quality of care they provide. The main objective of this survey was to explore the nature, characteristics and associations of stress in care home staff.

**Methods:**

Staff working in a stratified random sample of care homes within Wales completed measures covering: general health and wellbeing (SF-12); stress (Work Stress Inventory); job content (Karasek Job Content); approach to, and experience of, working with people living with dementia (Approaches to Dementia Questionnaire; and Experience of Working with Dementia Patients); and Productivity and Health Status (SPS-6). Multiple linear regressions explored the effects of home and staff characteristics on carers.

**Results:**

212 staff from 72 care homes completed questionnaires. Staff from nursing homes experienced more work stress than those from residential homes (difference 0.30; 95% confidence interval (CI) from 0.10 to 0.51; *P* < 0.01), and were more likely to report that their health reduced their ability to work (difference -4.77; CI -7.80 to -1.73; *P* < 0.01). Psychological demands on nurses were higher than on other staff (difference = 1.57; CI 0.03 to 3.10; *P* < 0.05). A positive approach to dementia was more evident in those trained in dementia care (difference 8.54; CI 2.31 to 14.76; *P* < 0.01), and in staff working in local authority homes than in the private sector (difference 7.75; CI 2.56 to 12.94; *P* < 0.01).

**Conclusions:**

Our study highlights the importance of dementia training in care homes, with a particular need in the private sector. An effective intervention to reduce stress in health and social care staff is required, especially in nursing and larger care homes, and for nursing staff.

**Trial registration:**

ISRCTN registry: ISRCTN80487202. Registered 24 July 2013

## Background

The (British) Alzheimer's Society estimates that there are currently 850,000 people living with dementia (PLWD) in the United Kingdom (UK) [1, 2]. One third of PLWD in the UK live in a care home, and research has shown that 80% of all people residing in care homes have a form of dementia or severe memory impairment [1]. Care homes and their staff therefore play a major role in managing dementia.

Depressive disorder, low mood, and anxiety are all commonly experienced by the carers of PLWD in their own homes [3–5]. Despite comprehensive literature on the mental health and wellbeing of family caregivers [5], there are relatively few studies of the mental health of care home workers looking after residents with dementia [6]. In a systematic review of stress in staff caring for PLWD, Pitfield and colleagues were able to identify just five studies, but these were either small samples or lacked robust evidence [7]. The largest of the included studies [8] found that higher carer stress correlated with a greater prevalence of behavioural and psychological symptoms (BPSD) in residents. However, in a study of staff working in nursing homes in Sydney, Australia, [9] found that, although staff reported that dealing with BPSD was the most difficult aspect of their work, these problems were not the main influences on nursing staff strain. Zimmerman and colleagues [10] examined stress in care workers from residential and nursing homes in the United States (US) and found that positive attitudes towards dementia and person-centred care correlated with job satisfaction.

In this study we have conducted a comprehensive nationally representative survey of care home staff across Wales who work with PLWD. The survey examines health, stress, wellbeing, job content, satisfaction and productivity in carers, and their approach to and experience of working with PLWD using well-validated measures. We have explored associations of these variables, notably with characteristics of care homes, staff education and training, job role, and experience of working with dementia.

## Methods

### Participants

We conducted this survey between February and October 2013 to inform our programme of research. The survey provided baseline data for a subsequent cluster randomised trial of mindfulness-based stress reduction for care home staff [11].

From a list of 374 care homes registered to care for PLWD with the Care and Social Services Inspectorate of Wales (a country within the UK) we excluded any facilities with fewer than 16 beds as the subsequent trial of mindfulness-based stress reduction required at least four staff from each participating home. We then used SPSS to sample 134 registered care homes at random. We first stratified homes by region (North, Mid, South East or South West Wales) and care sector (‘local authority’, ‘private’ or ‘voluntary’). We telephoned the managers of the 134 selected care homes to explain the study and invite them to participate. We explained to managers that, besides themselves, we needed three other staff to complete the questionnaires, namely one nurse (if the home had a nursing registration) and two other care staff providing direct personal care to residents. Of the 134 homes, 72 (54%) responded to the survey and a total of 212 staff volunteered to complete the survey questionnaires. As our purpose was not to compare responses between individual homes, we anonymised participating homes and their staff.

In the UK care homes are residential facilities that provide care for people in the short or long-term. ‘Local Authority homes’ are owned and run by local government authorities, whereas private homes are commercial facilities, and ‘voluntary sector homes’ are not run for profit but are typically owned by charities. Within all three sectors ‘residential homes’ provide accommodation, meals and personal care, but ‘nursing homes’ additionally employ registered nurses to provide care for more complex health needs.

### Questionnaires and measures

Our staff questionnaire included seven main sections: health and wellbeing, job satisfaction, job content, influence of health on job productivity, experience of working with dementia, attitudes towards dementia, and job stress. Where necessary, we obtained permissions to use questionnaires. We also collected demographic data including date of birth, gender, ethnicity, education and title of current job.

#### Health and well-being (SF-12)

We used Version 2 of the SF-12 to describe the health and wellbeing of the staff. This is a generic health-related quality-of-life instrument, originally developed as an even shorter version of the Short Form 36 [12]. It is a validated, precise and widely used instrument that measures eight aspects of general health. With each dimension rated from worst to best, the SF-12 generates two summary measures: the Physical Component Summary (PCS-12) and Mental Component Summary (MCS-12).

#### Andrew and Withey job satisfaction questionnaire (Satisfaction with Job Facets: SJF)

We measured global ‘job satisfaction’ by Andrew and Withey’s questionnaire [13]. This comprises five items each a seven-point scale from ‘delighted’ to ‘terrible’. We summed item scores to yield a total score between 7 and 35, with higher scores indicating greater job satisfaction. This scale has good internal consistency and construct validity.

#### Karasek Job Content Questionnaire (KJC)

This summarises the nature of jobs and their immediate context [14]. It comprises three sub-scales: decision latitude (with nine four-point items yielding scores between 9 and 36, showing the most latitude), psychological job demands (five four-point items with 20 showing the greatest demand) and social support in the work place (eight four-point items with 32 showing the best support).

#### Productivity & health status (SPS-6)

We used the Stanford Presenteeism Scale (SPS-6) to measure staff productivity adjusted for their health status [15]. Decreased presenteeism represents decreased productivity and impaired work quality. Each of the six five-point items explores staff’s ability to work within their work environment despite any health problems. Thus the SPS-6 total score ranges from 6 to 30, with 30 showing the greatest productivity. This instrument has good internal consistency and construct validity [15].

#### Experience of working with dementia patients (EWD)

This measure [16] assesses job satisfaction through 21 items, each scored from zero (not at all) to four (extremely). It yields six sub-scores: experience of feedback at work, experience of care organisation, satisfaction compared with own expectations, satisfactory contact with patients, satisfaction compared with expectations of others, and satisfaction with work environment. The instrument has good internal consistency, test-retest reliability and validity [16].

#### Approaches to Dementia Questionnaire (ADQ)

This measure [17] comprises 19 attitudinal items, each scored from 1 (strongly disagree) to 5 (strongly agree), and yields total score and two sub-scores - hope (8 items) and person-centredness (11 items). Additionally there are four sub-sub-scores: work events; caring for residents; workload and scheduling; and relations with supervisor.

#### Work Stress Inventory (WSI)

Zimmerman and colleagues examining stress in professional carers of nursing home residents with dementia [10] used a modified version of the original WSI [18]. Our study adopted this modified WSI, which comprises 30 stressors in four domains: work events (7 items); caring for residents (4 items); workload and scheduling (8 items); and relationship with supervisor (11 items). Each item is scored from 1 (never or not at all) to 5 (very often or very much). It yields a total score and four sub-scores ranging from 1 to 5, each equal to the average frequency of the contributing stressor scores [10]. Work events include: the use of unfamiliar equipment; overlapping responsibilities; being asked to do tasks without little or no training; making decisions about residents that should be made by another staff member; having little or no say in tasks assigned; having to make decisions on the spot; and being given responsibilities that are not part of the job. Workload and scheduling items include: working with inexperienced co-workers; not enough staff to care for residents properly; poor co-ordination of tasks in the work area; not getting time off when requested; working with workers who were not willing to try new things; not having enough time to discuss resident care issues with co-workers; having difficulty getting supplies and equipment; and too much paper work [10].

### Other variables

We collected data on the care homes including: type (with or without nursing), sector (private, Local Authority or voluntary), size (number of beds), number of beds registered for residents living with dementia, and number of permanent staff. We also collected data on staff including gender, age, ethnicity (white or other), job role (manager, senior carer, carer or care assistant, nurse), part- or full-time, average weekly working hours, trained in dementia care (yes or no), duration of work in care homes (in years), duration of work with PLWD (in years), and highest level of education or qualification (higher, supplementary, basic).

### Statistical analysis

For all these variables, we calculated summary statistics -- mean and standard deviation for continuous variables or percentages for categorical variables. We checked the reliability of all measurement scales comprising multiple Likert questions by assessing internal consistency through Cronbach’s alpha for respondents with no missing values. To estimate the effect of the socio-demographic and care home characteristics listed above on the seven total scores and 17 subscales also listed above, we used multiple linear regression with characteristics as independent variables. We checked all the assumptions underlying multiple regression by plotting residual errors and standardised residuals, checking variance inflation factors for multicollinearity, and conducting Durbin-Watson tests for correlated residuals. All these diagnostics fell within acceptable ranges for each of the 23 regression models. We analysed data by SPSS version 22 (SPSS Inc, Chicago, IL, USA).

## Results

Table 1 shows the characteristics of the 212 respondents from 72 care homes. Of these 32 (44%) homes provided nursing care; 53 (74%) were run privately, 12 (17%) by Local Authorities, and 7 (10%) by the voluntary sector. The mean number of residents in local authority homes was 32.4 (SD 7.5), private homes 39.3 (SD 22.9), and voluntary sector homes 46.3 (SD 23.8); these differences just failed to reach statistical significance (ANOVA; *P =* 0.062). The local authority employed 36 (32%) of residential care home staff, private homes 64 (57%), and the voluntary sector 12 (11%). Almost all staff working in nursing homes were in private facilities (94%); the remaining 6% were in the voluntary sector.

**Table 1:** Characteristics of care home staff

	N (%^a^) or Mean (SD)
Type of care facility	
Residential home	112 (52.8%)
Nursing home	100 (47.2%)
*Sector*	
Private	158 (74.5%)
Local Authority	36 (17.0%)
Voluntary	18 (8.5%)
Number of beds	38.7 (21.4)
Number of residents with dementia	19.7 (13.9)
Number of permanent staff	36.6 (24.4)
Male	29 (14.1%)
*Age*	44.0 (12.1)
<30 years	32 (15.5%)
30–39 years	35 (17.0%)
40–49 years	64 (31.1%)
50–59 years	56 (27.2%)
> = 60 years	19 (9.2%)
Ethnicity	
White	192 (91.9%)
Non-white	17 (8.1%)
Highest Education or Qualification achieved	
Low or none	58 (27.4%)
Medium	43 (20.3%)
Higher	111 (52.4%)
Current job title	
Manager	71 (35.0%)
Team leader or senior carer	39 (19.2%)
Carer or care assistant	71 (35.0%)
Nurse	22 (10.8%)
Full time	166 (81.0%)
Working hours per week	33.7 (10.4)
Trained in dementia care	174 (92.6%)
Work in care homes (years)	11.7 (8.55)
Work with people with dementia (years)	10.9 (8.45)

^a^After excluding missing values

Table 2 reports summary statistics and internal consistencies for the 23 scores arising from the seven main sections of the staff questionnaires. All but two of the 23 scores and sub-scores were positively skewed. All but two measurements achieved good reliability; however Cronbach’s alpha for job demands and satisfaction with the work environment both fell below 0.5.

**Table 2:** Work and welfare of care home staff^a^

Variables	Mean (SD)	Mini-mum	Maxi-mum	Internal consistency	
				No. of Items	Cronbach’s α
Health and wellbeing					
Total score (12–56)	47.1 (5.7)	28	55	12	0.84
Physical Component Subscore (PCS-12)	44.1 (4.0)	25.6	56.5	6	0.75
Mental Component Subscore (MCS-12)	46.2 (5.6)	29.9	57.0	6	0.82
Job satisfaction					
Total score (7–35)	27.0 (4.0)	15	35	5	0.87
Karasek Job Content					
Decision latitude subscore (9–36)	27.9 (3.6)	10	34	9	0.76
Job demands subscore (5–20)	13.3 (1.9)	8	20	5	0.35
Social support subscore (8–32)	24.2 (3.3)	12	32	8	0.82
Health status & employee productivity					
SPS-6 total score (6–30)	20.9 (5.8)	5	30	6	0.70
Experience of working with PLWD					
Total score (21–105)	85.1 (9.8)	58	105	21	0.89
Feedback at work subscore (6–30)	25.7 (3.5)	14	30	6	0.80
Care organisation subscore (3–15)	11.6 (2.2)	4	15	3	0.70
Against own expectations (3–15)	11.4 (2.3)	5	15	3	0.61
Contact with patient subscore (3–15)	13.2 (1.5)	7	15	3	0.65
Against others’ expectations (3–15)	12.5 (1.6)	8	15	3	0.51
Satisfaction with environment (3–15)	10.7 (2.0)	6	15	3	0.29
Approaches to Dementia					
Total score (19–95)	80.5 (8.5)	58	95	19	0.82
Hope subscore (8–40)	30.7 (5.5)	14	40	8	0.79
Person-centredness subscore (11–55)	50.1 (4.7)	30	55	11	0.77
Work Stress Inventory					
Total Score (1–5)	2.68 (0.42)	1.58	4.13	30	0.80
Work events subscore (1–5)	1.98 (0.67)	1	4.71	7	0.76
Caring for residents subscore (1–5)	3.10 (0.90)	1	5.00	4	0.74
Workload & scheduling subscore (1–5)	2.09 (0.78)	1	4.50	8	0.84
Relations with supervisor subscore (1–5)	3.41 (0.55)	1	5.00	11	0.72

^a^The denominator varies slightly across rows, as the number of missing values varies

To explore the relationship between individual instruments and sub-scales, we calculated Spearman’s rank correlation coefficients (ρ, non-parametric). There were many significant correlations between measures. We observed strong correlations (*ρ* > 0.5) between total scores of the EWD and SJF (*ρ* = 0.599), SJF and care organisation sub score of the EWD (ρ = 0.559), SJF and own expectation sub score of EWD (*ρ* = 0.558), SJF and KJC social support sub-scale (*ρ* = 0.554), SJF and workload schedule sub score of the WSI (*ρ* = -0.560).

Displayed within Table 3 are the twelve multiple linear regression models with all scores and sub-scores as dependent variables and all reported characteristics of care homes and their staff as predictors. Importantly, no characteristic had a significant effect on the total SF-12 score. However ‘experience of working with PLWD’ has a significant negative effect on the physical sub-score of the SF-12; each extra year of experience reduces the mean sub-score by 0.135 when all other variables are constant. Staff with basic education and qualifications report significantly greater job satisfaction scores than those from Higher Education.

**Table 3:** Multiple linear regression of welfare and work of care home staff on home and personal characteristics: coefficients and confidence intervals

	Health & well-being (SF-12)			Job satisfaction	Karasek Job Content			Productivity (SPS-6)	Experience working with PLWD	Approaches.to Dementia (ADQ)		
	Total	Physical	Mental	Total	Decision latitude	Psychological demands	Social support	Total	Total	Total	Hope	Person centredness
Type of home:												
Nursing	-2.44 (-5.9, 1.0)	0.93 (- 1.2,3.07)	-2.90 (-5.93,0.06)	-1.13 (-3.38, 1.12)	-1.16 (-3.01, 0.69)	0.04 (-1.06, 1.14)	-1.20 (-3.14, 0.72)	*-4.77* ^**^(-7.81,-1.72)	-1.18 (-6.72, 4.35)	1.97 (-2.53, 6.46)	0.58 (-2.32,3.47)	1.60 (-1.17,4.37)
Residential (reference)												
Sector:												
Local Authority	1.20 (-5.23,2.84)	1.67 (-0.789,4.14)	-3.06 (-6.52,0.39)	-0.51 (-3.11,2.09)	-1.04 (-3.17,1.1)	0.13 (-1.13,1.4)	-0.07 (-2.3,2.16)	-3.09 (-6.43,0.23)	-1.94 (-8.35,4.46)	*7.75* ^**^(2.55,12.93)	*4.75* ^**^(1.39,8.1)	*-3.20* ^*^(0.03,6.4)
Voluntary	-2.20 (-8.13,3.73)	2.04 (-1.57,5.66)	-3.15 (-8.21, 1.91)	0.04 (-3.77, 3.85)	-0.59 (-3.72, 2.55)	0.09 (-1.78, 1.95)	-1.26 (-4.53, 2.02)	-2.92 (-7.77, 1.92)	0.57 (-8.82, 9.95)	2.03 (-5.59, 9.65)	1.05 (-3.93, 6.03)	1.16 (-3.44,5.75)
Private (ref)												
Number of beds	0.014 (-0.12,0.15)	0.027 (-.056,0.11)	-0.040 (-0.15,0.076)	0.095 (-0.080,0.095)	-0.018 (-0.09,0.054)	0.032 (-0.011,0.075)	0.043 (-0.032,0.119)	0.079 (-0.033,0.19)	0.052 (-0.16,0.26)	-0.007 (-0.18,0.16)	0.003 (-0.11,0.11)	-0.005 (-0.11,0.105)
N residents with dementia	0.016 (-0.12,0.15)	-0.028 (-0.11,0.054)	0.030 (-0.085,0.145)	-0.023 (-0.11,0.064)	0.010 (-0.061,0.082)	0.008 (-0.035,0.050)	*-0.109* ^**^(-0.18,-0.034)	0.031 (-0.11,0.17)	0.046 (-0.17,0.26)	0.006 (-0.16,0.17)	-0.024 (-0.13,0.08)	0.033 (-0.074,0.13)
Care home staff:												
Male	1.26 (-2.99, 5.50)	-0.841 (-3.41.7)	0.11 (-3.65,3.86)	1.52 (-1.2,4.25)	0.3 (-1.94,2.54)	0.59 (-0.74,1.92)	1.41 (-0.94,3.75)	-1.54 (-4.99,1.91)	2.69 (-4.05,9.42)	0.78 (-4.67,6.23)	0.38 (-3.11,3.86)	0.32 (-2.96,3.6)
Age (years)	-0.027 (-0.15, 0.10)	0.051 (-0.026, 0.129)	-0.063 (-0.17, 0.046)	-0.055 (-0.13, 0.028)	0.003 (-0.064, 0.07)	*-0.061* ^**^(-0.101, -0.021)	-0.047 (-0.11, 0.023)	0.032 (-0.072, 0.13)	0.019 (-0.18, 0.22)	-0.050 (-0.21, 0.11)	0.020 (-0.085, 0.12)	-0.075 (-0.17, 0.025)
Current job title:												
Nurse	-0.37 (-5.26,4.52)	1.11 (-1.9,4.09)	-0.83 (-3.34,4.99)	0.16 (-2.99,3.3)	-2.06 (-4.64,0.52)	*1.57* ^*^(0.02,3.1)	1.39 (-1.31,4.09)	1.15 (-3.06,5.36)	-6.24 (-13.97,1.49	-2.92 (-9.2,3.36)	-2.48 (-6.59,1.64)	-0.29 (-4.11,3.52)
Senior carer	-1.62 (-6.4,3.1)	0.97 (-1.9,3.8)	-1.87 (-5.9,2.16)	-2.54 (-5.59,0.52)	*-3.9* ^**^(-6.35, -1.34)	-0.19 (-1.67,1.3)	-0.26 (-2.87,2.36)	-0.03 (-4,3.93)	-3.82 (-11.34,3.71	-4.90 (-10.98,1.18)	-2.95 (-7.02,1.11)	-1.89 (-5.6,1.82)
Carer	-2.43 (-8.1,3.3)	0.33 (-3.15,3.8)	-0.06 (-4.93,4.8)	-3.31 (-6.98,0.35)	*-4.98* ^**^(-7.99,-1.96)	-0.90 (-2.69,0.89)	*-3.19* ^*^(-6.34, -0.03)	0.71 (-3.97,5.39)	-5.46 (-14.8,3.88)	*-7.51* ^*^(-14.84, -0.18)	-3.48 (-8.46,1.51)	- 3.43 (-7.88,1.02)
Manager (ref)												
Working hours/week	0.026 (-.15,.19)	-0.099 (-0.203,0.006)	0.037 (-0.11,0.18)	0.017 (-0.094,0.12)	0.020 (-0.070,0.11)	0.009 (-0.045,0.063)	0.030 (-0.065,0.12)	-0.13 (-0.27,0.008)	0.13 (-0.14,0.403)	-0.006 (-0.22,0.21)	-0.022 (-0.16,0.12)	0.034 (-0.101,0.16)
Training in dementia	-1.83 (-6.7,3.01)	0.53 (-2.43,3.48)	-3.28 (-7.41,0.85)	-0.94 (-4.05,2.17)	-0.096 (-2.65,2.46)	-0.304 (-1.82,1.22)	0.99 (-1.68,3.67)	-1.97 (-5.91,1.96)	3.93 (-3.73,11.58)	*8.54* ^**^(2.31,14.75)	*4.15* ^*^(0.15,8.13)	*4.43* ^*^(0.608,8.24)
Work with dementia (yrs)	0.094 (-0.105,0.292)	*-0.14* ^*^(-0.26, -.014)	0.083 (-0.087,0.25)	-0.014 (-0.14,0.11)	-0.010 (-0.11,0.095)	-0.002 (-0.064,0.061)	-0.017 (-0.12,0.092)	*0.24* ^**^(0.079,0.404)	0.103 (-0.21,0.41)	0.12 (-0.13,0.37)	0.053 (-0.11,0.21)	0.066 (-0.090,0.22)
Basic education	-2.51 (-2.57,7.59)	1.25 (-1.8,4.35)	-2.37 (-6.7,1.96)	*3.44* ^*^ (0.15,6.71)	0.06 (-2.74,2.62)	1.26 (-0.34,2.85)	1.87 (-0.94,4.67)	0.87 (-3.29,5.04)	2.04 (-6.35,10.42	1.56 (-4.96,8.09)	-0.19 (-4.51,4.12)	1.72 (-2.29,5.73)
Supplementary education	2.56 (-1.76,6.88)	0.27 (-2.37,2.9)	1.28 (-2.4,4.96)	2.56 (-0.23,5.35)	1.84 (-0.44,4.12)	*1.91* ^**^(0.55,3.27)	2.36 (-0.02,4.75)	-0.31 (-3.86,3.24)	6.91 (-0.19,14.01	2.58 (-2.96,8.13)	0.36 (-3.43,4.14)	2.09 (-1.25,5.43)
Higher Education (ref)												

**P < 0.01; *P < 0.05

Only characteristics that are significant in either Table 3 or 4 are presented

Regression analysis of the Karasek Job Content Questionnaire (Table 3) indicates that staff with primary care worker roles reported significantly less support from their supervisors in the work place and less decision latitude than home managers. Mean decision latitude was also significantly lower for senior carers than managers. Additionally, nursing staff experienced greater psychological demands than managers. Productivity as measured by the Stanford Presenteeism Scale indicated that staff of nursing homes scored a mean 4.8 points fewer than staff of residential homes; and each extra year of working with people living with dementia increases the productivity score by 0.24.

The Approaches to Dementia Questionnaire (ADQ) shows that local authority staff have a more positive attitude than private care home staff, scoring 7.8 more in total and 4.8 more on the hope subscale, but 3.2 less on the person-centredness subscale. As we expected, staff with formal dementia care training scored more in total (by 8.5), more on the hope subscale (by 4.2) and more on the person-centered subscale (by 4.4). Care assistants scored less on average than other types of staff on the ADQ, and significantly less than managerial staff.

The Work Stress Inventory (WSI) generally revealed strong relationships (Table 4). Nursing homes are more stressful than other homes on: the work events subscale (by 0.51 points on average; *P* <0.01); the workload and scheduling subscale (by 0.49; *P* <0.05); and the total score (by 0.30; *P* <0.01). Furthermore each extra bed reduces scores on the caring for residents subscale by 0.02 (*P* <0.05); staff with basic education and qualifications report 0.75 less stress from workload and scheduling (*P* < 0.05); and each extra working hour per week increases relationship with supervisor sub-scores by 0.02 (*P* < 0.05). Total WSI scores are on average 0.44 lower (*P* < 0.01) for those with basic education and qualifications than those from Higher Education.

**Table 4:** Multiple linear regression of Work Stress Inventory of care home staffs on home and personal characteristics: coefficients and confidence intervals

	Work Stress Inventory (WSI)				
	Total	Work events	Caring for residents	Workload & scheduling	Relationship with supervisor
Type of home:					
Nursing	*0.30* ^**^(0.09,0.51)	*0.51* ^**^(0.15,0.85)	0.48 (-0.03,0.98)	*0.49* ^*^(0.08,0.91)	-0.03 (-0.34,0.28)
Residential (reference group)					
Sector:					
Local Authority	0.18 (-0.05,0.42)	0.39 (-0.01,0.79)	0.27 (-0.31,0.85)	0.29 (-0.18,0.76)	-0.05 (-0.41,0.3)
Voluntary	0.14 (-0.21,0.49)	*0.96* ^**^(0.37,1.55)	-0.21 (-1.07,0.65)	0.12 -0.57,0.82)	-0.23 (-0.75,0.29)
Private (ref)					
Number of beds	-0.005 (-0.013,0.003)	-0.010 (-0.023,0.004)	*-0.021* ^*^(-0.040,0.001)	-0.009 (-0.025,0.007)	0.006 (-0.006,0.018)
N residents with dementia.	0.002 (0.006,0.010)	-0.001 (-0.023,0.004)	0.012 (-0.008,0.031)	-0.005 (-0.021,0.011)	0.005 (-0.007,0.017)
Care home staff:					
Male	-0.040 (-0.29,0.21)	0.11 (-0.31,0.53)	-0.27 (-0.89,0.34)	0.06 (-0.44,0.56)	-0.12 (-0.49,0.25)
Age (yrs)	-0.002 (-0.010,0.005)	-0.00 (-0.01,0.01)	-0.008 (-0.026,0.011)	-0.002 (-0.017,0.013)	-0.001 (-0.012,0.01)
Current job title:					
Nurse	0.05 (-0.23,0.34)	-0.23 (-0.71,0.26)	0.17 (-0.54,0.88)	0.16 (-0.42,0.73)	0.12 (-0.31,0.55)
Senior carer	-0.19 (-0.46,0.09)	-0.22 (-0.69,0.25)	-0.54 (-1.22,0.15)	-0.14 (-0.69,0.42)	-0.07 (-0.49,0.34)
Carer	0.04 (-0.29,0.37)	0.19 (-0.38,0.75)	-0.39 (-1.21,0.44)	0.33 (-0.34,1)	-0.11 (-0.61,0.39)
Manager (ref)					
Working hours/wk	0.010 (0.000,0.020)	0.002 (-0.015,0.019)	0.006 (-0.019,0.031)	0.007 (-0.014,0.027)	*0.019* ^*^(0.004,0.034)
Training in dementia	-0.078 (-0.36,0.21)	0.002 (-0.48,0.48)	0.035 (-0.67,0.74)	-0.131 (-0.704,0.44)	-0.13 (-0.55,0.29)
Work with dementia (yrs)	-0.003 (-0.014,0.009)	-0.003 (-0.022,0.017)	0.003 (-0.026,0.032)	-0.017 (-0.04,0.007)	0.006 (-0.012,0.010)
Basic education	*-0.44* ^**^(-0.73, -0.13)	-0.47 (-0.97,0.03)	-0.46 (-1.19,0.28)	*-0.75* ^*^(-1.35, -0.15)	-0.17 (-0.62,0.27)
Supplementary education	0.19 (-0.44,0.06)	-0.27 (-0.70,0.16)	-0.08 (-0.70,0.55)	0.36 (-0.86,0.15)	-0.05 (-0.42,0.33)
Higher Education (ref)					

**P < 0.01; *P < 0.05

Only characteristics that are significant in either Table 3 or 4 are presented

## Discussion

This comprehensive, nationally representative survey of residential and nursing home facilities caring for people living with dementia explored the characteristics of care home staff that affected seven dimensions of their welfare and performance: health and wellbeing; job satisfaction; job content; productivity; their experience of, and approaches to, dementia care; and work-related stress. Our key findings were:

- Staff working in nursing homes experienced greater levels of stress; this was primarily associated with work events, workload and scheduling rather than personal care. Compared with residential only homes, staff in nursing homes were also more likely to report that their health adversely influenced work performance and experiences as measured by the Stanford Presenteeism Scale. The demands on nursing staff were significantly greater than on other carers in this environment, though care workers felt least supported. More beds also contributed to the stress of caring for residents.
- A positive approach to dementia was particularly evident in those who had received training in dementia care, and those working for local authorities rather than the private sector.
- Carers with fewer formal qualifications experienced greater job satisfaction and less stress.
- The longer staff had worked in a residential care environment, the worse the effect on physical health, but not psychological.

### Results in context

Another important finding of our survey was low mean SF-12 scores: 44 on the physical component, 46 on the mental component, and 47 overall. The SF-12 is widely used to measure perceived general health and wellbeing, and these scores are all lower than the corresponding scores reported by the Utah Department of Health [19]: 51 on the physical component, 52 on the mental component, and 50 in total. Although there are no norms for the SF-12 in the UK, there is conflicting evidence whether US norms for the SF-36 are valid in British studies [20, 21]. Professional carers in our study rated their health better than family caregivers from the English National Survey [22]. Although there is no comparative British data for the WSI, care home staff scored more overall and on most subscales than staff working in US care facilities [10], suggesting greater levels of stress in our participants. Nursing homes appeared particularly stressful in our study; working in a nursing home has previously been recognised as a physically and mentally demanding occupation at greater risk of work- and stress-related diseases [23]. Similarly, Ejaz et al [24] found that nursing homes reported less job satisfaction amongst direct care workers than two other types of long term care.

We found that the demands on nursing staff were significantly higher than on other carers. A report describing the broad and multi-faceted roles and responsibilities of such nurses has also highlighted this [25]. Nurses also have a central role in supporting and supervising care staff and undertake administrative and managerial functions. A shortage of staff may also affect the demands made on nursing staff. The Care Quality Commission (CQC) in England found that 20% of nursing homes do not have enough staff on duty to ensure safe care for residents [26]. They suggested that there are fewer career development opportunities for nurses working outside the National Health Service (NHS), and pressure to increase the numbers of acute nurses within the NHS could contribute to the shortages. They also reported that the National Minimum Data Set for Social Care [26] showed that nurses working in care homes have the highest annual turnover rates (32%) of all social care roles. All these factors may account for higher stress levels and demands, particularly on nurses in nursing homes.

We also found that larger homes contributed to the stress of caring for residents. McCracken & Fitzwater [27] found that small units were positively associated with increased supervision and interaction between staff and residents with dementia. Annerstedt [28] found that staff in small group-living units reported greater competence, more knowledge in dealing with dementia, and greater satisfaction than those in traditional nursing homes. Pekkarinen [29] reported that large units increased time pressure on employees and reduced quality of life of residents. Demands on staff were lower and social support from co-workers was higher in small group-living homes than in larger traditional nursing homes [30]; satisfaction was also higher and burnout lower in these small homes. The CQC [26] also found that homes with fewer beds tended to perform better than larger units.

We found positive approaches to dementia particularly evident in staff who had received training in dementia care, and staff working in local authority homes rather than the private sector. This finding is similar to those of others investigating the role of education and training for staff caring for PLWD [31–34]. Good care for PLWD relies on staff having knowledge of dementia care as well as a positive attitude [10]. Travers et al [35] found that participation in dementia education and training within 12 months significantly predicted a more positive attitude.

Carers with fewer formal qualifications experienced greater job satisfaction and less stress. This finding is consistent with others who have shown that Higher Education leads to lower job satisfaction [36, 37]. However this conflicts with a study that found that less education was associated with high job strain in residential dementia care [38].

### Strengths & limitations

The particular strengths of our survey are: the random, nationally representative sample of care homes, covering all health care sectors and home types; and the wide scope of the survey with comprehensive and validated measures of staff welfare, attitudes towards dementia care, and performance. Limitations include the moderate response rate from homes (54%), and the self-selection of staff within these homes to complete survey questionnaires. Though the survey recorded carer characteristics, it could not measure residents’ symptoms and characteristics that are known to be important. We also had to rely on home managers to estimate numbers of people living with dementia in each home.

## Conclusions

Our study has emphasized the importance of staff training, and highlighted a particular need for this within private care homes in the UK. This supports the World Health Organisation’s recommendation of enhanced workforce education and training programmes on dementia and long-term care [39]; and the Care Quality Commission (CQC) regulation proposing that staff must receive the training, support, supervision, professional development and appraisals necessary for them to fulfil their role and responsibilities [26]. Research has shown that, across healthcare, stress has negative effects on staff and the workplace that can lead to high sickness, high absenteeism, high turnover, lower productivity, low job satisfaction, and burnout [40–43]. These reduce the quality and effectiveness of patient or resident care [44]. Our work highlights the need for effective interventions to reduce stress among health and social care staff, especially nursing staff working in care homes, notably larger homes and nursing homes. These conclusions are particularly pertinent when the care home sector has been reported as in crisis [45], and when the latest CQC annual review reported that adult social care services in the UK were at ‘tipping point’ [46]. Moreover research in partnership with UNISON, the principal trade union in British healthcare, reported that staff working in care homes were not being provided with sufficient training, particularly in dementia care [47].

## Abbreviations

ADQ: Approaches to Dementia Questionnaire
BPSD: Behavioural and Psychological Symptoms of Dementia
CQC: Care Quality Commission
CSSIW: Care and Social Services Inspectorate of Wales
EWD: Experience of Working with Dementia
KJC: Karasek Job Content Questionnaire
MCS: Mental Component Summary
NHS: National Health Service
PCS: Physical Component Summary
PLWD: People living with dementia
SD: Standard Deviation
SJF: Satisfaction with Jobs Facets
SPS: Stanford Presenteeism Scale
UK: United Kingdom
US: United States of America
WSI: Work Stress Inventory
